# Primary Cardiac Fibroma in an Adult

**DOI:** 10.1155/2015/713702

**Published:** 2015-09-20

**Authors:** Samuel H. Cho, Timothy Fritz, Lynn J. Cronin, Stephen D. Cohle

**Affiliations:** ^1^Michigan State University College of Human Medicine, Grand Rapids, MI, USA; ^2^Spectrum Health Frederik Meijer Heart & Vascular Institute, Grand Rapids, MI, USA; ^3^Spectrum Health Department of Pathology, Grand Rapids, MI, USA

## Abstract

Cardiac fibromas are benign primary tumors composed of connective tissue and fibroblasts. These uncommon tumors are primarily found in the pediatric population, and their prevalence among the adult population is exceedingly rare. We report a case of an adult with nonspecific symptoms, who was subsequently found to have a solitary mass located in the left ventricle. This case highlights an unusual finding in an adult who through various imaging modalities, surgical excision, and immunohistological analysis was found to have a cardiac fibroma.

## 1. Introduction

Cardiac fibromas are benign primary tumors composed of connective tissue and fibroblasts. These uncommon tumors are primarily found in the pediatric population [1], and their prevalence among the adult population is exceedingly rare. We present a case of an adult with nonspecific symptoms, who was subsequently discovered to have a solitary mass located in the left ventricle suggestive of a cardiac fibroma.

## 2. Case Presentation

A 60-year-old woman presented with an acute onset of fatigue, shortness of breath, chest pain, and palpitations, following an episode of bronchitis. The patient also had a 7-pound weight gain over the past 1-2 weeks. She was found to have moderate pericardial effusion, and during preliminary imaging for echocardiographic guided pericardiocentesis a mass was noted on the epicardial surface of the left ventricle. The pericardiocentesis was deferred, and the patient underwent further diagnostic imaging.

**Figure 1:**
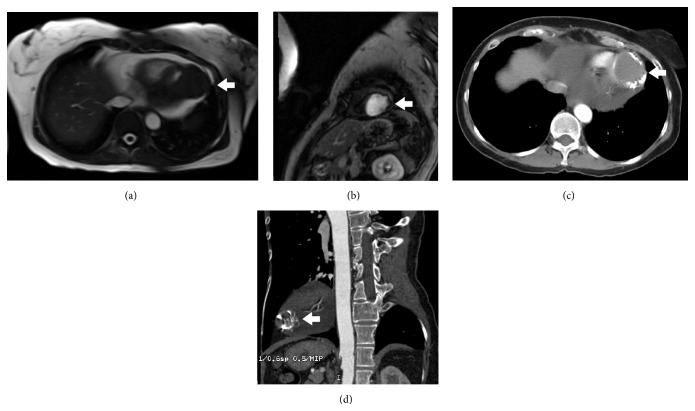
MRI imaging revealing the cardiac fibroma attached to the left ventricle (white arrow) surrounded by pericardial effusion (a and b). CT imaging revealing cardiac fibroma with peripheral calcifications (c and d).

An echocardiogram conclusion was a large, well-circumscribed epicardial mass on the left ventricular surface of the heart, not consistent with a left ventricular pseudoaneurysm. The mass was solid, and it appeared to sit on top of the normal myocardium. Cardiac MRI with and without contrast showed an approximate 4 cm broad based attachment to the inferolateral myocardium of the left ventricle apex (Figures 1(a) and 1(b)). The mass was T1 isointense to muscle with areas of T2 hypointensity, suggestive of fibrosis [2]. Noncontrast chest CT revealed a low attenuation mass with prominent calcification at the left ventricular apex with moderately sized pericardial effusion (Figures 1(c) and 1(d)).

**Figure 2:**
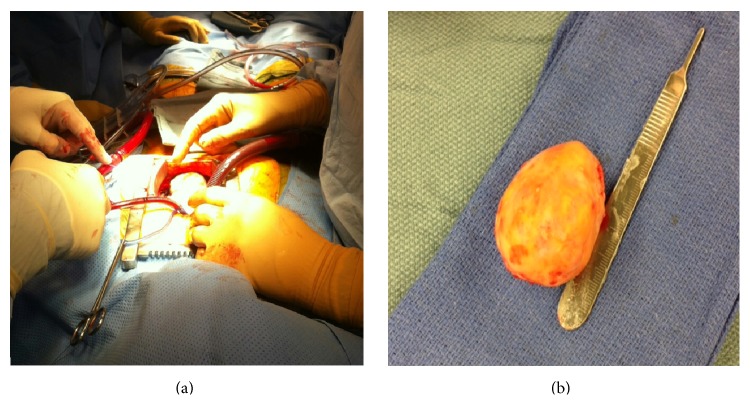
Gross image of the cardiac mass measuring 5.5 × 4.0 × 3.5 cm.

Complete surgical resection was carried out under cardiopulmonary bypass. The operative finding was 5.5 × 4.0 × 3.5 cm tan-brown colored mass at the posterior lateral portion of the heart near the apex (Figure 2). The tumor was found to be in close proximity to the left anterior descending artery. The edge of the tumor was discerned and was not invading into the myocardium. Closure was made with a patch, and postprocedure transesophageal echocardiogram demonstrated well-preserved left ventricular and right ventricular function.

**Figure 3:**
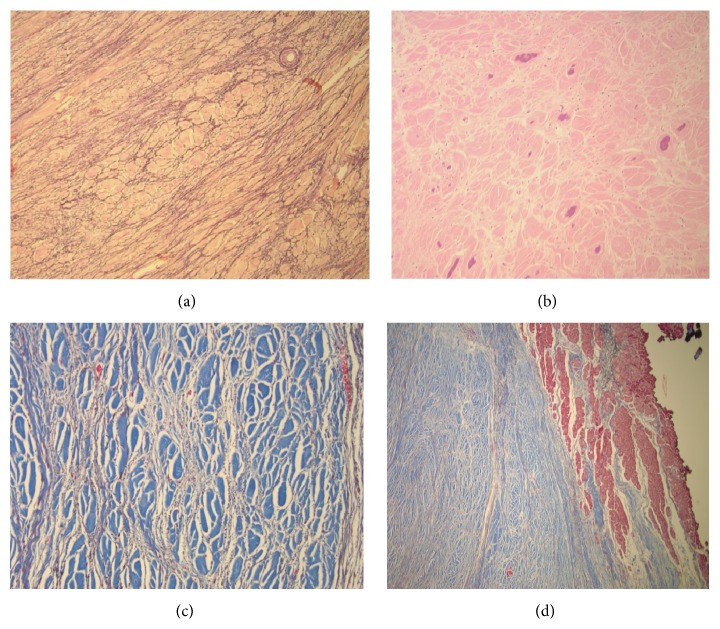
Histologic slides of the cardiac fibroma revealing numerous elastic fibers (a) and calcific stippling (b). Trichrome staining (c and d) revealed well demarcation from the interface of the tumor with the normal myocardium (red) and showed groups of fascicles of collagen (blue) surrounded by black-staining elastic fibers.

Sectioning revealed tan-white, rubbery, whorled, calcified cut surfaces (Figure 2(b)). Neither hemorrhage nor necrosis was present and the mass was sent for histopathological examination. Microscopically, a trichrome stain highlighted a sharp demarcation between the fibroma (blue) and normal myocardium (red) (Figure 3(a)). At higher power, the fibroma consisted of fascicles of collagen (Figure 3(b)) which, with an elastic stain, were surrounded by elastic fibers (Figure 3(c)). The mass also displayed purple calcific stippling (H&E stain) (Figure 3(d)). The patient had an uncomplicated hospital course and was discharged home on postoperative day 5.

## 3. Conclusions

This case highlights an extremely unusual finding in an adult who through various imaging modalities, surgical excision, and immunohistological analysis was found to have a cardiac fibroma in the left ventricle. While cardiac fibromas are rare solitary lesions that lack metastatic potential, they are the most common neoplastic cause of life-threatening arrhythmias and even sudden death. Largely dependent on the size and location of the tumor, patients may present with clinical manifestations relating to conduction defects, ventricular arrhythmias, congestive heart failure, and hemodynamic compromise [3, 4].

In our patient, the presenting symptoms were very nonspecific, and there were no signs of any conduction abnormalities, congestive heart failure, or hemodynamic instability. Incidental findings of a pericardial effusion and a mass on an echocardiogram prompted further investigation by cardiac MRI and CT. Surgical resection appears to be a safe and effective method for treatment.
